# A ‘dynamic adder model’ for cell size homeostasis in *Dictyostelium* cells

**DOI:** 10.1038/s41598-021-92700-3

**Published:** 2021-07-02

**Authors:** Masahito Tanaka, Toshiko Kitanishi-Yumura, Shigehiko Yumura

**Affiliations:** 1 Graduate School of Sciences and Technology for Innovation, Yamaguchi University, Yamaguchi, 753-8512 Japan; 2 Present Address: Laboratory of Physics and Cell Biology, Department of Chromosome Science, National Institute of Genetics, 1111 Yata, Mishima, Shizuoka 411-8540 Japan

**Keywords:** Cell growth, Cell division, Cell growth

## Abstract

After a cell divides into two daughter cells, the total cell surface area of the daughter cells should increase to the original size to maintain cell size homeostasis in a single cell cycle. Previously, three models have been proposed to explain the regulation of cell size homeostasis: sizer, timer, and adder models. Here, we precisely measured the total cell surface area of *Dictyostelium* cells in a whole cell cycle by using the agar-overlay method, which eliminated the influence of surface membrane reservoirs, such as microvilli and membrane wrinkles. The total cell surface area exponentially increased during interphase, slightly decreased at metaphase, and then increased by approximately 20% during cytokinesis. From the analysis of the added surface area, we concluded that the cell size was regulated by the adder or near-adder model in interphase. This adder model is not caused by a simple cell membrane addition, but is more dynamic due to the rapid cell membrane turnover. We propose a ‘dynamic adder model’ to explain cell size homeostasis in interphase.

## Introduction

What determines the size of cells is a large question for cell biologists^1–3^. Generally, the cellular size is the smallest immediately after cell division (at birth); the daughter cells grow during the cell cycle and become the largest just before the next division, in which cells maintain their original cell size. Cell size homeostasis has been mainly studied in yeasts and bacteria, because their cell shapes are relatively simple, and their size can be easily measured^4–6^. Studies of these cells have shown that they regulate the growth rate and the cell cycle in such a way that larger cells at birth divide earlier than smaller ones, and vice versa^7,8^.

There are three models to explain the regulation of cell size homeostasis: sizer, timer, and adder models. The sizer model has been reported for fission yeast, whose cells divide after growing to a certain size^9^. The timer model has been reported for *Caulobacter crescentus*, whose cells divide after a certain time^10^. Adder models have been reported for *Escherichia coli, Bacillus subtilis*, and budding yeast, whose cells divide after a certain amount of cell size (cell volume, cell surface area, among others) is added, independently of their initial size^4,11,12^. As bacteria and yeasts have cell walls, the regulation of their cell size includes the regulation of cell walls. Although measuring the size of mammalian cells is difficult because they have an irregular shape, a few studies have measured the cell volume by using fluorescence exclusion or microfabricated channels and showed that their cell size is regulated by adder model or near-adder model^13,14^. The most recent study showed that cell size of mouse epidermal stem cells in vivo is regulated by sizer model^15^. Additionally, recent study has reported that the growth rate does not decrease although the larger animal cells which range from diploid to massively polyploid decrease surface-to-volume ratio^16^, suggesting that the cell size is independent of the initial size.

Because the cell volume tends to change depending on the intracellular hydrostatic pressure, the total cell surface area could be a more reliable parameter of cell size^13,14,17,18^. The precise measurement of the total cell surface area is more difficult because of small microvilli or wrinkles on the cell surface in animal cells, which occupy 21–130% of the apparent cell surface area^19,20^. Recently, we developed a method to precisely measure the total cell surface area, via which cells are flattened by overlaying with an agar block, which expands the small protrusions and wrinkles present on the cell surface^21^.

Here, by using the agar-overlay method, we examined the dynamics of the total cell surface area in the whole cell cycle of *Dictyostelium* cells, a model organism for cell growth, cell division, and cell migration. From the analysis of the added surface area, the cell surface area at birth, and the generation time, we found that the cell size homeostasis during interphase of *Dictyostelium* cells is regulated by the adder or near-adder model. However, the adder model was not as simple as previously discussed, being considerably dynamic due to the rapid turnover of the cell membrane. We propose a ‘dynamic adder model’ to explain cell size homeostasis.

## Results

### Long period-observation of cells under an agar-overlay

**Figure 1:**
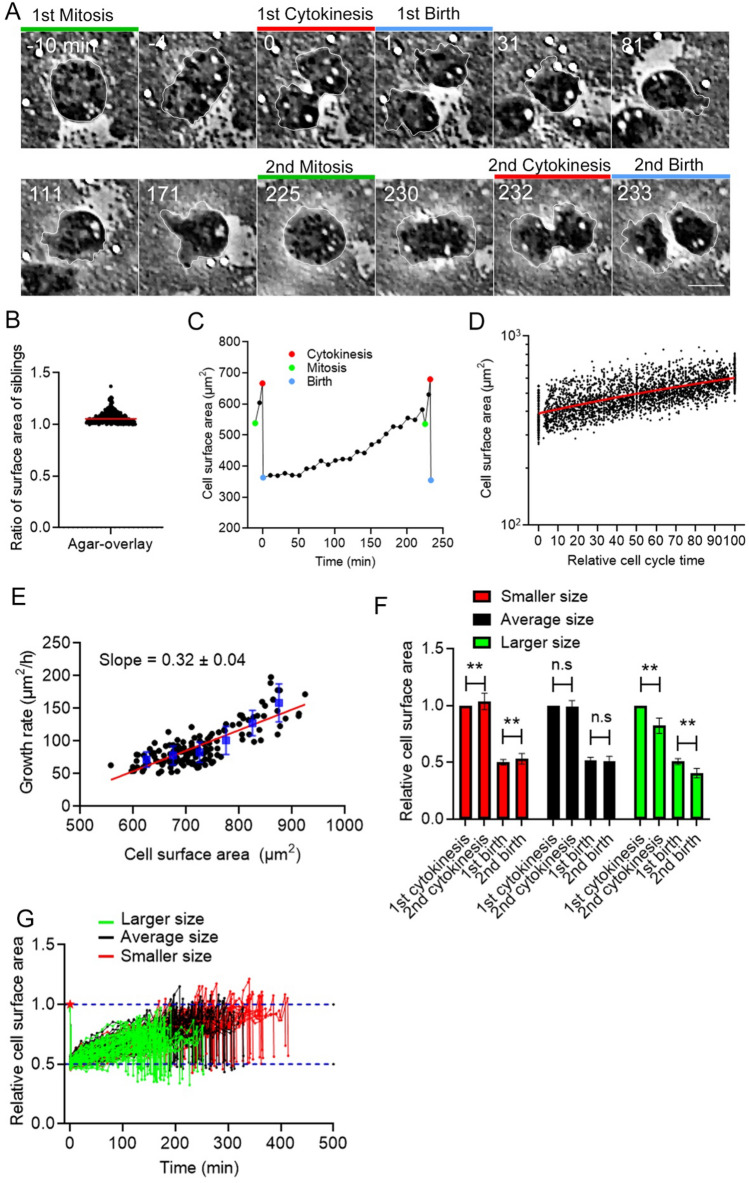
Control of the total cell surface area in a whole cell cycle. (**A**) Time course of phase-contrast images of *Dictyostelium* cells in a whole cell cycle. The green lines show the mitotic phase, the red lines show cytokinesis, and the blue lines show the cells immediately after cell division (at birth). The cell was outlined with white lines. Scale bar, 10 μm. (**B**) Ratios of the surface area between sibling cells (larger cells/smaller cells) The cells divide almost evenly with a small deviation (7.02% ± 5.94%, n = 227). (**C**) Representative time course of the total cell surface area in a whole cell cycle. The colored marks indicate the same events shown in (**A**). (**D**) Time courses of total cell surface area in the logarithmic scale from birth (0) until immediately before the next mitosis (100) in multiple cells (n = 147). The relative cell cycle time (only interphase) is normalized. The red line shows a result by linear regression. (**E**) The instantaneous growth rate versus the cell surface area immediately before cytokinesis. The blue vertical lines show the binned mean ± SD. A linear regression line is shown in red, which supports the exponential growth of cell surface area. (**F**) Comparison of the relative cell surface area between the 1st and 2nd cytokinesis and at the first and second birth in larger (> 450 µm^2^, green), average (400 ± 50 µm^2^, black), and smaller sized cells (< 350 µm^2^, red). Data are presented as the mean ± SD. **P < 0.001; ns, not significant; P > 0.05. (**G**) Time courses of the relative cell surface area of individual cells from the first division (red asterisk) to the second division (n = 147). The blue dotted lines show 0.5 and 1.0 of the relative cell surface area, respectively.

Previously, we developed a method to precisely measure the total cell surface area, in which cells are flattened by overlaying with an agar block, which expands the small protrusions and wrinkles of the cell surface^21,22^. To examine the dynamics of the total cell surface area in a whole cell cycle, cells were observed under an agar-overlay for a long period (up to 12 h). In our early experiments, we used an agar block containing a nutrient medium (HL5), but the cell size was gradually reduced, presumably because cells did not fully uptake the nutrient from the agar block^23^. Since *Dictyostelium* cells grow by phagocytosing bacteria in a natural habitat, cells were observed in the presence of live bacteria (*Escherichia coli*) under an agar-overlay (Fig. 1A). In these conditions, the doubling time was approximately 4 h (3.87 ± 1.15 h, n = 147), which is consistent with the previous observation in a suspension culture in the presence of bacteria^24^. It has been reported that in a confined space, such as a microchannel, mammalian cells tend to asymmetrically divide, creating siblings with different sizes^13,14^. However, *Dictyostelium* cells are normally and symmetrically divided under an agar-overlay. Figure 1B shows the differences in the total cell surface area between the siblings (7.02% ± 5.94%, n = 227). Therefore, we established a method to observe cells under an agar-overlay for a long period.

### Dynamics of total cell surface area in a whole cell cycle

Figure 1C shows the typical time course of a single cell surface area in a whole cell cycle. The total cell surface area increases after cytokinesis (red mark on the left), subtly decreases at mitosis (green mark on the right), increases by approximately 20% during cytokinesis, and decreases after cell division (blue mark on the right). The changes in the cell surface area during mitosis and cytokinesis were consistent with our previous observations^21^. *Dictyostelium* cells have a prolonged G2 phase that accounts for over 90% of the cell cycle, and M and S phases account for approximately 10%^25^. Thus, from birth until immediately before the next mitosis, the cells are almost in G2 phase. Figure 1D shows a graph of total cell surface area in the logarithmic scale versus the relative cell cycle time in multiple cells (n = 147), to assess whether the cell size increased exponentially or linearly. The relative cell cycle time was normalized from the birth until immediately before the next mitosis. The graph was linear in a logarithmic scale, indicating that the cell size increases exponentially in G2 phase. Figure 1E shows a graph of the instantaneous growth rate versus the cell surface area immediately before cytokinesis, indicating that larger cells grow at higher rate than smaller cells, resulting in the exponential growth of the cell surface area.

When the cells were divided into three populations: larger (> 450 µm^2^, green), average (400 ± 50 µm^2^, black), and smaller size (< 350 µm^2^, red) (Fig. 1F), the size ratio of the 2nd cytokinesis (immediately before division)/the 1st birth in the average-size cell population was close to 2 (1.92 ± 0.09), and the cell size returned to the original at the 2nd birth. However, the larger cells significantly decreased in size after the second cell division compared with after the first cell division. On the other hand, smaller cells significantly increased in size after the second cell division compared with after the first cell division. Figure 1G shows the time courses of individual cells, suggesting that larger cells (green) divide much earlier than smaller cells (red). These results suggest that *Dictyostelium* cells have a cell size homeostasis mechanism that makes cells of a deviated size return to the average size.

### Cell surface area in *Dictyostelium* cells is regulated by an adder or near-adder model

There are three models to explain the regulation of cell size homeostasis: sizer, timer, and adder models. These models can be assessed by quantifying the total cell surface areas at birth and immediately before the 2nd cell division, the added surface area between them, and the generation time^7,8,26^.

**Figure 2:**
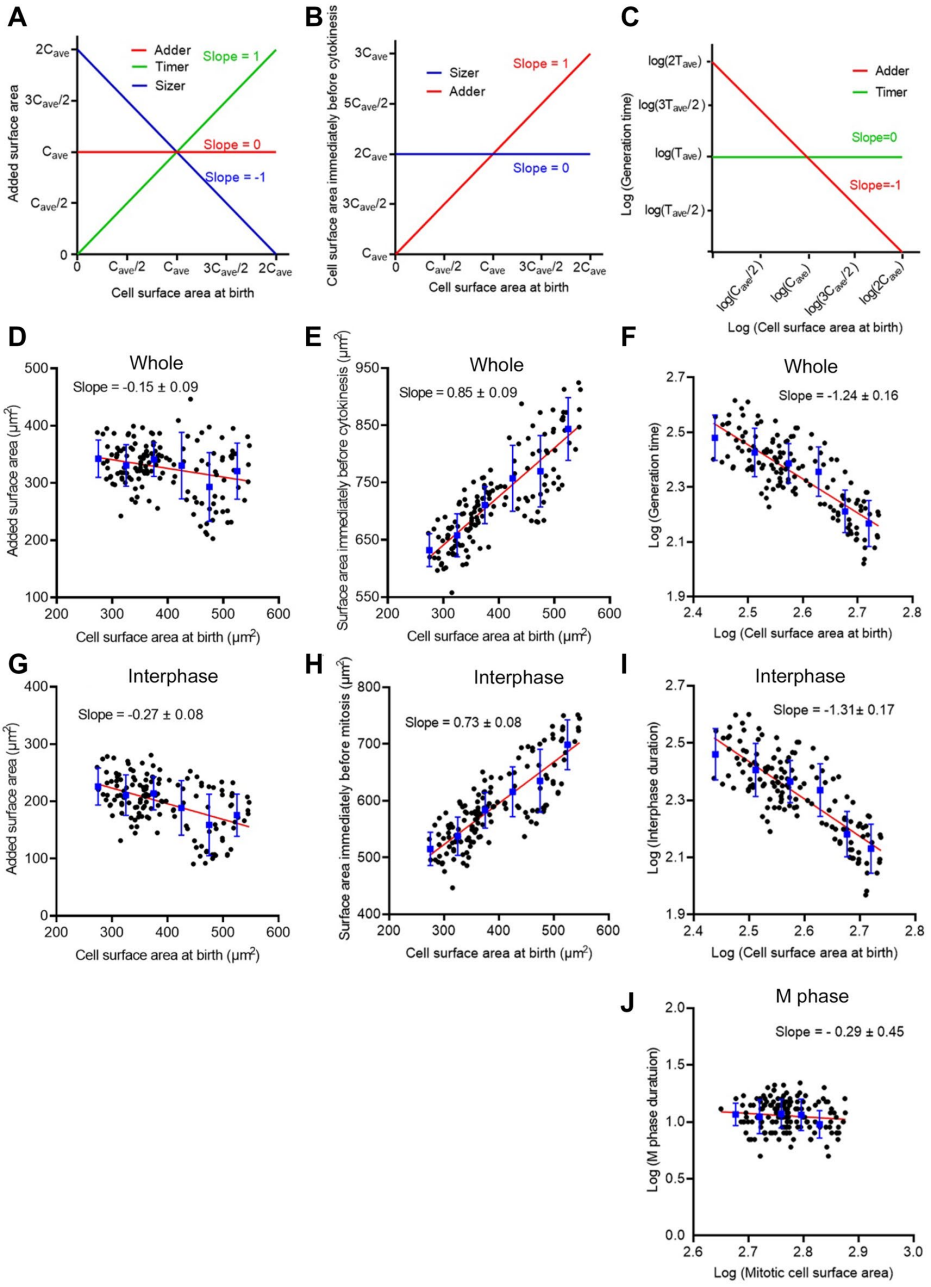
Cell surface area in *Dictyostelium* cells is regulated by an adder or near-adder model. (**A**–**C**) Ideal graphs to assess the three models to explain the regulation of cell size homeostasis. (**A**) The added surface area during cell cycle versus the cell surface area at birth. (**B**) The cell surface area immediately before cytokinesis versus the cell surface area at birth. (**C**) The logarithmic generation time versus the logarithmic cell surface area at birth. C_ave_ and T_ave_ show the average cell surface area at birth and the average generation time, respectively. (**D**–**F**) Graphs of actual data in a whole cell cycle (Whole). (**G**–**I**) Graphs of actual data between the first cell division and immediately before mitosis (Interphase). (**J**) A graph of actual data during cell division (M phase). In graphs **D**–**J**, the red lines show results by linear regression. The blue vertical lines show the binned mean ± SD.

Figure 2A–C show ideal graphs to assess the three models. Figure 2A shows graphs of the added surface area during a single cell cycle versus the cell surface area at birth. If the slope is + 1, the model should be the timer model when cells show exponential growth (green). If the slope is 0, the model should be the adder model (red). If the slope is − 1, the model should be the sizer model (blue). Figure 2B shows graphs of the cell surface area immediately before cytokinesis versus the cell surface area at birth. If the slope is + 1, the model should be the adder model. If the slope is 0, the model should be the sizer model. Figure 2C shows graphs of the logarithmic generation time versus the logarithmic cell surface area at birth. If the slope is -1, the model should be the adder model (red). If the slope is 0, the model should be the timer model (green). These graphs were plotted as described in the Methods section.

Figure 2D shows actual plots for *Dictyostelium* cells with respect to the added surface area during the whole cell cycle versus the cell surface area at birth, indicating that the slope was − 0.15. Figure 2E shows actual plots of the cell surface area immediately before cytokinesis versus the cell surface area at birth, indicating that the slope was 0.85. Figure 2F shows actual plots of the logarithmic generation time versus the logarithmic cell surface area at birth, indicating that the slope was -1.24. These results suggest that the cell size in *Dictyostelium* is regulated by an adder or near-adder model.

### The duration of mitotic phase is constant independently of the cell size

Here, these model assessments were conducted during the whole cell cycle, including both interphase and mitotic phases. Previous model assessments in other cells have been conducted during the interphase, but not during the mitotic phase. Figure 2G–I show that similar assessments were conducted only for the interphase. The slopes of the individual assessments were almost similar to those of the whole cell cycle, suggesting that the cell size is regulated by an adder or near-adder model in the interphase.

Next, we examined the cells in the mitotic phase. Figure 2J shows the logarithmic cell division time in the mitotic phase versus the logarithmic surface area. The cell division time in the mitotic phase was the duration from cell rounding to final abscission. The duration of mitotic phase was constant independently of the cell size, implying that there is a clock to determine the duration of mitotic phase.

### The cell surface area is maintained by a dynamic balance between exocytosis and endocytosis

**Figure 3:**
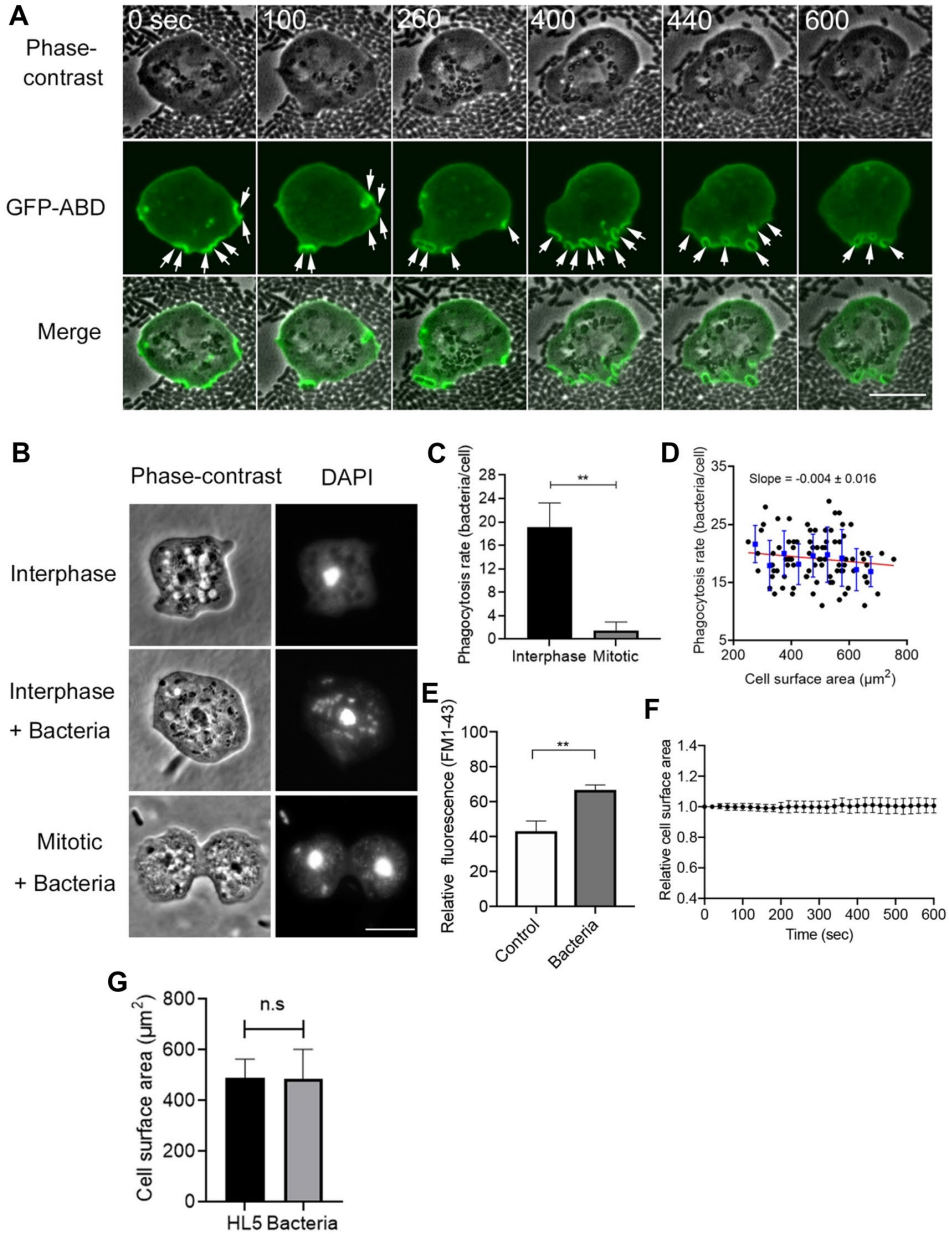
The cell surface area is maintained by a dynamic balance between exocytosis and endocytosis. (**A**) Representative time courses of phase-contrast, fluorescence, and merge images of a live cell expressing GFP-ABD in the presence of bacteria. Arrows indicate bacteria being internalized by the cells. Scale bar, 10 µm. (**B**) Representative phase-contrast and fluorescence images of cells stained with DAPI in the presence and absence of bacteria. Many bacteria were found in interphase cells, but a few bacteria were found in mitotic cells. Scale bar, 10 μm. (**C**) The number of bacteria within interphase and mitotic cells. Data are presented as the mean ± SD. **P < 0.001; n ≥ 80 for each. (**D**) The phagocytosis rate for 10 min versus the cell surface area at birth. The blue vertical lines show the binned mean ± SD. Linear regression lines are shown in red (n = 87). (**E**) Quantitative analysis of the membrane uptake in the presence and absence of bacteria (for 10 min). Cells were stained with FM1-43 and measured using fluorescence spectrophotometry in each condition. Data are presented as the mean ± SD. **P < 0.001, three different experiments. (**F**) A time course of the total cell surface area in the presence of bacteria (n = 28). (**G**) The total surface area in the presence and absence of bacteria. Data are presented as the mean ± SD (n ≥ 87 for each). ns, not significant.

The increase in the cell surface area is not caused by the simple addition of the membrane via exocytosis. In the present conditions, cells constantly endocytosed bacteria, suggesting that cells internalized massive membranes. Figure 3A shows the phase-contrast and fluorescence images of cells expressing ABD (actin binding domain of *Dictyostelium* filamin), an F-actin marker, which were observed in the presence of bacteria. Actin filaments were localized at the phagocytic cups (arrows), suggesting that the cells vigorously engulf the bacteria. The number of internalized bacteria for 10 min was examined by staining with DAPI (4’,6-diamidine-2’-phenylindole dihydrochloride) after fixation (Fig. 3B,C). Approximately 19 (19.1 ± 4.1, n = 87) bacteria were found in individual cells, which is consistent with previous observations^27,28^. Figure 3D shows actual plots of phagocytosis rate for 10 min versus the cell surface area at birth, indicating that phagocytosis rate is independent of cell size. Incidentally, only approximately 1.5 (1.53 ± 1.38, n = 88) bacteria were found in mitotic cells (Fig. 3C), indicating that phagocytosis was significantly reduced in the mitotic phase, which has not been reported in *Dictyostelium* cells, although the macropinocytosis and clathrin-mediated endocytosis were reported to be reduced during mitosis^21^.

To examine the amount of internalized cell membrane during phagocytosis, the fluorescence intensities of the cells in the presence of FM1-43, which emits fluorescence when inserted into the lipid bilayer, were measured using fluorescence spectrophotometry. Since the cell membranes of bacteria were also stained with FM dye, the fluorescence intensities of bacteria were subtracted from those of *Dictyostelium* cells with bacteria in their interior. Figure 3E shows that membrane uptake was significantly increased when cells internalized bacteria for 10 min. Figure 3F shows the time course of the total cell surface area in the presence of bacteria, indicating that it was almost constant, in spite of the vigorous internalization of cell membranes. In addition, we compared the total surface areas in the presence of bacteria and in HL5 medium. The total surface area was measured 10 min after cells were mixed with bacteria (Fig. 3G). The surface area in the presence of bacteria was 486.2 ± 116.2 μm^2^ (n = 87) and that of the HL5 medium was 491.5 ± 72.0 μm^2^ (n = 105), which were not significantly different.

Therefore, exocytosis should compensate for the cell membrane that was lost by phagocytosis.

Together, the total cell surface area is maintained by a dynamic balance between exocytosis and endocytosis.

## Discussion

Here, we precisely measured the total cell surface area in a whole cell cycle of *Dictyostelium* cells by using the agar-overlay method. We found that the total cell surface area increased exponentially during interphase without any specific time point of increase. Larger cells grew at higher rate than the smaller cells and divided much earlier than the smaller cells (Fig. 1E,G). From the analysis of added surface area, we concluded that the cell size homeostasis during interphase of *Dictyostelium* cells is regulated by the adder or near-adder model.

**Figure 4:**
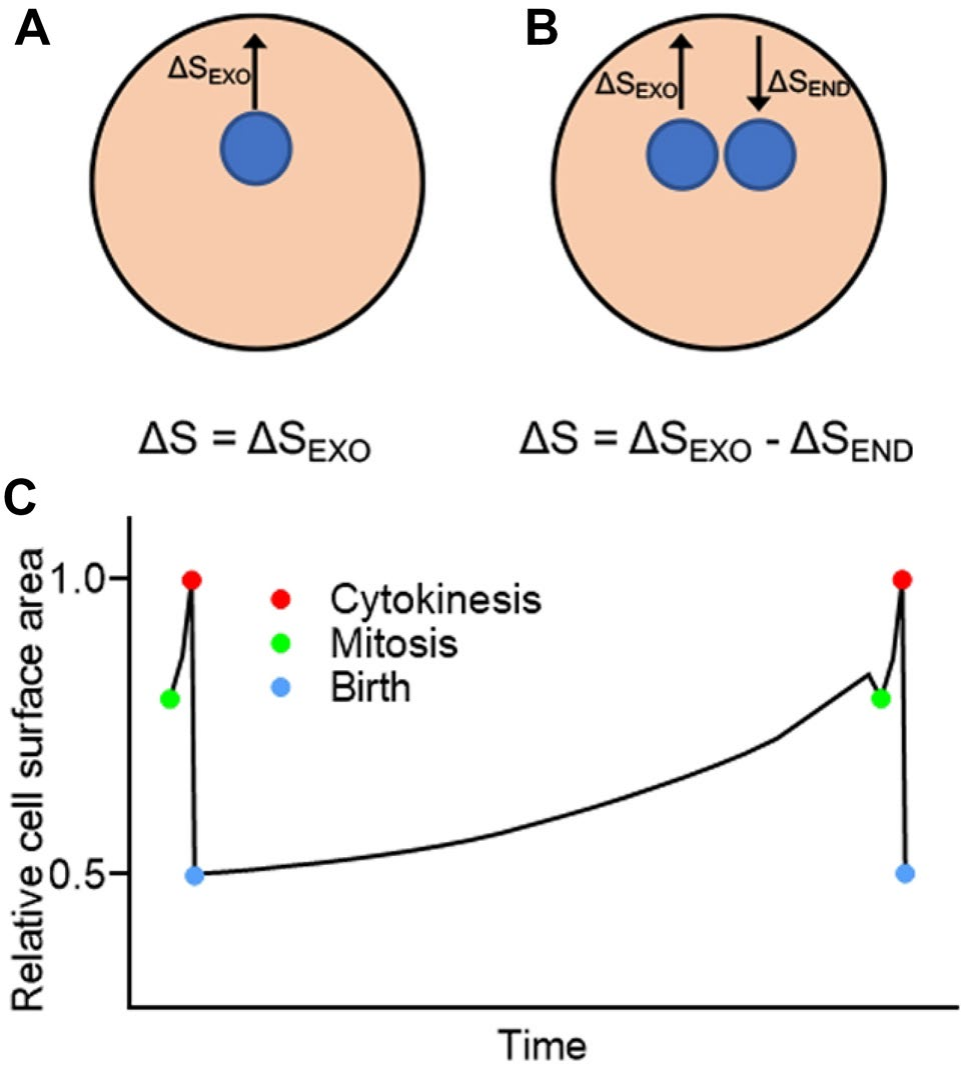
Dynamic adder model. (**A**) A previous adder model considered only the added surface area without taking into account the turnover of cell membrane. Here, ΔS is the total added surface area, and ΔS_EXO_ is the added surface area due to exocytosis. (**B**) Since the cell membrane rapidly turns over, the total added surface area (ΔS) should be determined by subtracting the amount of membrane uptake by endocytosis (ΔS_END_) from that of membrane supply by exocytosis (ΔS_EXO_). (**C**) A summary of the dynamics of the total cell surface area in a whole cell cycle of *Dictyostelium* cells.

In the adder model, cells divide after a certain amount of cell membrane or cell volume is added^4,11,12^. The previous adder model did not take into account the cell membrane turnover but only its addition (Fig. 4A). However, the cell membrane rapidly turns over in migrating cells during interphase^22^. In addition, if cells phagocytose 20 bacteria per 10 min, the internalized cell membrane area is about 180 µm^2^, which is equivalent to 30–60% of the total cell membrane. If the duration of interphase is 220 min, the whole cell membrane should be refreshed 7–13 times. However, the total cell surface area only doubles, suggesting that exocytosis compensates for the amount of membrane lost via endocytosis. Therefore, the conventional adder model should be revised to ‘dynamic adder model’, where the added surface area should be determined by subtracting the amount of membrane uptake via endocytosis from that of membrane supply via exocytosis (Fig. 4B).

Exocytosis should exceed endocytosis to increase the cell size, and cells must always monitor their cell size and regulate it via endocytosis and exocytosis. As a simple explanation for this mechanism, they may monitor the added amount of cell membrane components. As reported in animal cells, cells may begin to divide by sensing the ratio between lipids in the cell membrane and cytoplasm^29^.

Additionally, the cortical tension may contribute to the monitoring. Previous studies have reported that exocytosis and endocytosis are regulated by the cortical tension^30–32^. In macrophages, exocytosis is facilitated by increasing the cortical tension via actin polymerization at phagocytic cups^33^. In *Dictyostelium* cells, actin filaments are also localized at the phagocytic cups as shown in Fig. 3A, which may increase the cortical tension and facilitate exocytosis. In addition, the cortical tension may increase as the cell size increases, which may trigger the preparation for cell division.

We found that the duration of mitotic phase is almost constant regardless of cell size, indicating that there is a clock to determine the duration of mitotic phase. The mitotic phase should be strictly and intricately regulated to maintain genome integrity. Disordered mitotic phase inhibits normal spindle formation and causes failure of cell division^34,35^. Recent study has reported that the duration of mitotic phase in animal cells is remarkably constant regardless of varying cell cycle length, which is regulated by cdk1-cyclin B positive feedback^36^. When perturbed by cyclin B overexpression, *Dictyostelium* cells are arrested in mitotic phase^37^. In *Caenorhabditis elegans* embryo and fission yeast, it has been reported that the duration of mitotic phase is maintained constant by changing the speed of the furrow constriction and the spindle elongation in a manner dependent on the cell size^38,39^. We previously showed that cell surface area is strictly regulated during cytokinesis, and an approximately 20% increase of cell surface area is essential for the constriction of the cleavage furrow^21^. In present study, the total cell surface area did not return to the original only during interphase, suggesting that the increases of cell surface area both during interphase and mitotic phase are essential for the cell size homeostasis. How the cell size is regulated during mitotic phase should be scrutinized in more detail in future.

Figure 4C summarizes the dynamics of the total cell surface area in the cell cycle of *Dictyostelium* cells. We propose a ‘dynamic adder model’ to explain cell size homeostasis in the interphase. To explain the constant increase in cell surface area during the interphase, exocytosis should slightly exceed endocytosis in the dynamic turnover of the cell membrane. The molecular mechanism underlying the precise regulation of cell size remains to be clarified.

## Methods

### Cell Culture

*Dictyostelium discoideum* wild type (AX2) cells were cultured in a plastic dish at 22 °C in HL5 medium (1.3% bacteriological peptone, 0.75% yeast extract, 85.5 mM D-glucose, 3.5 mM Na_2_HPO_4_ 12H_2_O, 3.5 mM KH_2_PO_4_, pH 6.3). An extra-chromosomal expression vector of GFP-ABD (actin binding domain of *Dictyostelium* filamin) was transformed into cells via electroporation or laserporation, as described previously^40,41^. Transformed cells were selected in HL5 medium supplemented with 10 μg/mL G418 (Wako, Osaka, Japan). For the shaking culture, the cells were cultured in conical flasks (100 mL) containing 20 mL of HL5 medium or 20 mL of a Na/K-phosphate buffer containing *Escherichia coli* (*E. coli, B/r*) at 22 °C in a reciprocal shaker at 150 rpm. To synchronize the cell cycle to increase the number of mitotic cells, cells were cultured at 10 °C for 16 h and treated with 100 µM thiabendazole (TB) at 22 °C for 3.5 h^42^. *E. coli* was cultured in HL5 medium in suspension at 37 °C and washed with a 15 mM Na–K phosphate buffer (pH 6.3) using centrifugation.

### Microscopy

*Dictyostelium* cells and bacteria were placed in a glass-bottom dish and overlaid with an agarose block^43^. After the agar-overlay, the cells were observed under an optical sectioning fluorescence microscope (Deltavision, GE Healthcare Life Science, United Kingdom). The images were acquired over time and stitched from 12 images stacks arranged in a 4 × 3 grid (total area is 824 × 612 μm.) every 1 min.

To observe the fixed cells, the agar-overlaid cells were fixed by immersing in ethanol containing 1% formaldehyde at -17 °C, as described previously^43^. Fixed cells were stained with DAPI (Sigma-Aldrich, Tokyo, Japan) and tetramethyl rhodamine (TRITC)-phalloidin (Sigma-Aldrich) and observed under a fluorescence microscope (TE 300, Nikon, Japan) equipped with regular UV and TRITC filter sets.

Fluorescence images of live cells expressing GFP-ABD in the presence of *E. coli* were acquired using a confocal microscope (LSM510, Zeiss, Germany) at time intervals of 20 s. The total cell surface areas were calculated from the cell outline and thickness by using ImageJ software (http://rsbweb.nih.gov/ij), as described previously^21^. All images were processed and analyzed using ImageJ software.

### Assessment of three models for cell size homeostasis

Three models for cell size homeostasis (sizer, timer, and adder models) were assessed based on the slopes in Fig. 2A–C and on previous reports^7,8,26^. When cell size homeostasis is regulated by perfect sizer, timer, or adder models, the relationship between the added surface area during a single cell cycle (ΔS) versus the cell surface area at birth (x) for the three models is the following:

1 $${\text{adder model}}:\quad \Delta {\text{S}} = {\text{ C}}_{{{\text{ave}}}}$$

2 $${\text{sizer model}}: \quad \Delta {\text{S }} = {\text{2C}}_{{{\text{ave}}}} {-}{\text{ x}}$$

If cells show exponential growth,

3 $${\text{timer model}}:\quad \Delta {\text{S }} = {\text{ x}}$$

where C_ave_ is the average size at birth. As shown in Fig. 2A, the slope is 0 for the adder model, − 1 for the sizer model, and + 1 for the timer model.

The relationship between the cell surface area immediately before cytokinesis (S_cytokinesis_) and the cell surface area at birth (x) for the sizer and adder models is the following:

4 $${\text{adder model}}:\quad {\text{ S}}_{{{\text{cytokinesis}}}} = {\text{ x }} + {\text{ C}}_{{{\text{ave}}}}$$

5 $${\text{sizer model}}:{\text{ }}\quad {\text{S}}_{{{\text{cytokinesis}}}} = {\text{ 2C}}_{{{\text{ave}}}}$$

As shown in Fig. 2B, the slope is + 1 for the adder model and 0 for the sizer model.

The relationship between the generation time and the cell surface area at birth for the timer and adder models is the following:

6 $${\text{timer model}}: \quad {\text{generation time }} = {\text{T}}_{{{\text{ave}}}}$$

7 $${\text{adder model}}:\quad {\text{generation time }} = {\text{ C}}_{{{\text{ave}}}} {\text{T}}_{{{\text{ave}}}} /{\text{cell surface area at birth}}$$

where T_ave_ is an average generation time.

To obtain the slopes of linear lines, both sides of the equations were converted to logarithms (T: logarithmic generation time, x: logarithmic cell surface area at birth), as follows:

8 $${\text{timer model}}:{\text{ }}\quad {\text{T }} = {\text{ logT}}_{{{\text{ave}}}}$$

9 $${\text{adder model}}:{\text{ }}\quad {\text{T }} = {\text{ logC}}_{{{\text{ave}}}} + {\text{ logT}}_{{{\text{ave}}}} {-}{\text{ x}}$$

As shown in Fig. 2C, the slope is 0 for the timer model and − 1 for the adder model.

### Uptake of cell membranes

The uptake of cell membranes was measured by staining cells with 10 µM FM1-43 (Thermo Fisher Scientific, Tokyo, Japan), a fluorescent lipid analog. Since the nutrient medium hampered the staining, the cells were stained after the medium had been exchanged with 15 mM Na/K phosphate buffer (pH 6.4) containing 0.1 M sorbitol. Sorbitol was used to suppress the activity of contractile vacuoles^44^. Ten minutes after staining, cells were washed twice with a Na/K phosphate buffer containing 15 mM sodium azide to suppress exocytosis. The fluorescence intensities (excitation at 470 nm and emission at 570 nm) were measured using a fluorescence spectrophotometer (F-2500, Hitachi High-Technologies, Corp., Tokyo, Japan).

To quantify the uptake of the cell membrane during phagocytosis, *Dictyostelium* cells (4 × 10^6^ cells/mL) were mixed with *E. coli* cells (1 × 10^8^ cells/mL) in Na/K phosphate buffer. Since the cell membrane of *E. coli* was also stained, the fluorescence intensity of *E. coli* cells was subtracted from that of *Dictyostelium* cells containing bacteria.

### Statistical analysis

Statistical analysis and linear regression analysis were conducted using GraphPad Prism 8 (GraphPad Software, Inc., San Diego, CA, United States) (https://www.graphpad.com). Data are presented as mean ± SD and analyzed using unpaired two-tailed Student’s t-test or one-way ANOVA with Tukey’s multiple comparison test.

## Data Availability

All relevant data are available from the authors on reasonable request.
